# The Impact of Ligand Carboxylates on Electrocatalyzed Water Oxidation

**DOI:** 10.1021/acs.accounts.1c00298

**Published:** 2021-08-17

**Authors:** Biswanath Das, Ahibur Rahaman, Andrey Shatskiy, Oscar Verho, Markus D. Kärkäs, Björn Åkermark

**Affiliations:** † Department of Organic Chemistry, Arrhenius Laboratory, Stockholm University, Svante Arrhenius väg 16C, SE-10691 Stockholm, Sweden; ‡ Division of Organic Chemistry, Department of Chemistry, KTH Royal Institute of Technology, SE-10044 Stockholm, Sweden; § Department of Medicinal Chemistry, Drug Design and Discovery, Biomedicinskt Centrum BMC, Uppsala University, Husargatan 3, SE-75123 Uppsala, Sweden

## Abstract

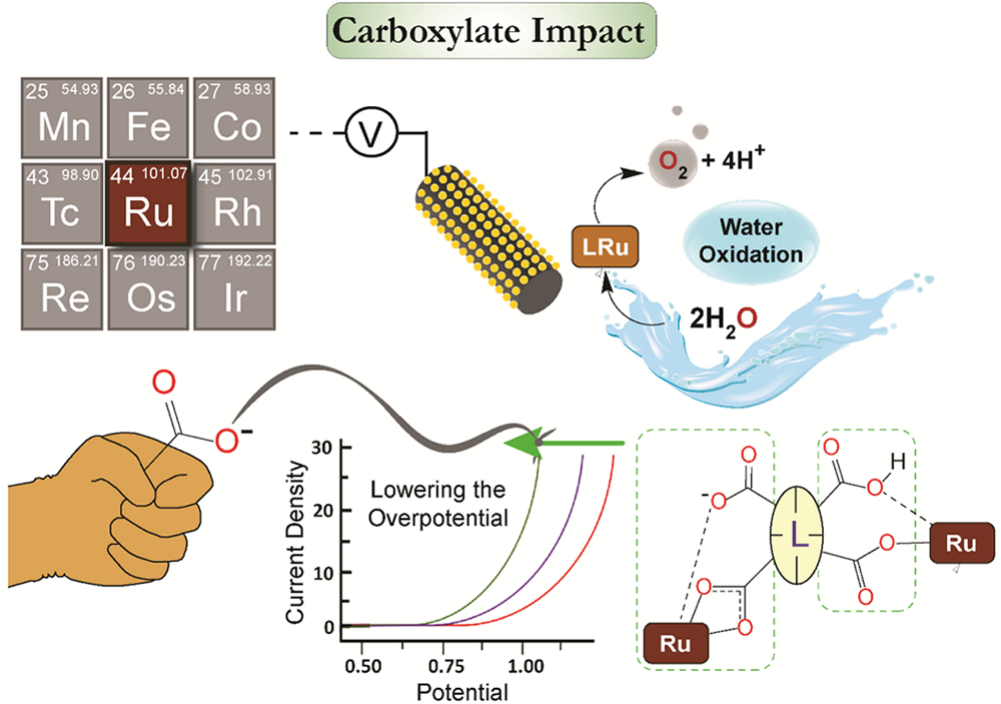

Fossil fuel shortage and severe climate changes due to global warming have prompted extensive research on carbon-neutral and renewable energy resources. Hydrogen gas (H_2_), a clean and high energy density fuel, has emerged as a potential solution for both fulfilling energy demands and diminishing the emission of greenhouse gases. Currently, water oxidation (WO) constitutes the bottleneck in the overall process of producing H_2_ from water. As a result, the design of efficient catalysts for WO has become an intensively pursued area of research in recent years. Among all the molecular catalysts reported to date, ruthenium-based catalysts have attracted particular attention due to their robust nature and higher activity compared to catalysts based on other transition metals.

Over the past two decades, we and others have studied a wide range of ruthenium complexes displaying impressive catalytic performance for WO in terms of turnover number (TON) and turnover frequency (TOF). However, to produce practically applicable electrochemical, photochemical, or photo-electrochemical WO reactors, further improvement of the catalysts’ structure to decrease the overpotential and increase the WO rate is of utmost importance. WO reaction, that is, the production of molecular oxygen and protons from water, requires the formation of an O–O bond through the orchestration of multiple proton and electron transfers. Promotion of these processes using redox noninnocent ligand frameworks that can accept and transfer electrons has therefore attracted substantial attention. The strategic modifications of the ligand structure in ruthenium complexes to enable proton-coupled electron transfer (PCET) and atom proton transfer (APT; in the context of WO, it is the oxygen atom (metal oxo) transfer to the oxygen atom of a water molecule in concert with proton transfer to another water molecule) to facilitate the O–O bond formation have played a central role in these efforts.

In particular, promising results have been obtained with ligand frameworks containing carboxylic acid groups that either are directly bonded to the metal center or reside in the close vicinity. The improvement of redox and chemical properties of the catalysts by introduction of carboxylate groups in the ligands has proven to be quite general as demonstrated for a range of mono- and dinuclear ruthenium complexes featuring ligand scaffolds based on pyridine, imidazole, and pyridazine cores. In the first coordination sphere, the carboxylate groups are firmly coordinated to the metal center as negatively charged ligands, improving the stability of the complexes and preventing metal leaching during catalysis. Another important phenomenon is the reduction of the potentials required for the formation of higher valent intermediates, especially metal-oxo species, which take active part in the key O–O bond formation step. Furthermore, the free carboxylic acid/carboxylate units in the proximity to the active center have shown exciting proton donor/acceptor properties (through PCET or APT, chemically noninnocent) that can dramatically improve the rate as well as the overpotential of the WO reaction.

## Key References

- XuY.; FischerA.; DuanL.; TongL.; GabrielssonE.; ÅkermarkB.; SunL.Chemical and Light-Driven Oxidation of Water Catalyzed by an Efficient Dinuclear Ruthenium Complex. Angew. Chem., Int. Ed.2010, 49, 8934–893710.1002/anie.20100427820941720.^1^*The cooperative impact of the relative cis orientation of two ruthenium centers encapsulating the carboxylate containing noninnocent ligand framework in electro- and photochemical WO was reported. Two well-characterized ruthenium complexes (cis and trans) with very similar ligand scaffolds were investigated.*
- KärkäsM. D.; ÅkermarkT.; JohnstonE. V.; KarimS. R.; LaineT. M.; LeeB.-L.; ÅkermarkT.; PrivalovT.; ÅkermarkB.Water Oxidation by Single-Site Ruthenium Complexes: Using Ligands as Redox and Proton Transfer Mediators. Angew. Chem., Int. Ed.2012, 51, 11589–1159310.1002/anie.20120501823023986.^2^*Evaluation of WO activities of two structurally similar benzimidazole containing mononuclear ruthenium complexes showed encouraging influence from the carboxylate group in the ligand framework in improving TON and TOF for WO, induced by mild one-electron oxidant [Ru(bpy)_3_]^3+^ (bpy = 2,2′-bipyridine)*.
- DasB.; EzzedinlooL.; BhadbhadeM.; BucknallM. P.; ColbranS. B.Strategic Design of a Ruthenium Catalyst for Both CO_2_ Reduction and H_2_O Oxidation: The Electronic Influence of the Co-Ligands. Chem. Commun.2017, 53, 10006–1000910.1039/c7cc06294j28835944.^3^*The possibility of both pH and redox dependent ring opening involving phenanthroline carboxylate ligand that is directly bonded to the ruthenium center was discussed. The phenanthroline carboxylate unit in the catalyst works as a combined redox and proton-transfer mediator*.
- ShatskiyA.; BardinA. A.; OschmannM.; MatheuR.; Benet-BuchholzJ.; ErikssonL.; KärkäsM. D.; JohnstonE. V.; Gimbert-SuriñachC.; LlobetA.; ÅkermarkB.Electrochemically Driven Water Oxidation by a Highly Active Ruthenium-Based Catalyst. ChemSusChem2019, 12, 2251–22623075932410.1002/cssc.201900097.^4^*The carboxylate group dangling near the metal center was involved in proton-coupled electron transfer (PCET) and intramolecular atom proton transfer (i-APT) to achieve the active form [Ru^IV^(O)(mcbp)(py)_2_] [mcbp^2–^ = 2,6-bis(1-methyl-4-(carboxylate)-benzimidazol-2-yl)pyridine] in mild conditions and lower the activation barrier for the O–O bond formation.*

## 1 Introduction and Brief Historical Background

**Scheme 1:**
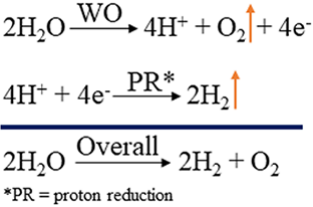
Schematic Representation of the Overall Process of H_2_ Generation from H_2_O

Water oxidation (WO) and proton reduction have attracted global attention with the promise to deliver clean and renewable fuel in the form of H_2_ from water (Scheme 1). In the overall process of water splitting, WO, which is a multiproton and multielectron step, stands out as the limiting step.

We and others have investigated several ruthenium catalysts over the years with the aim to bring down the activation barrier for WO.^5−7^ In this regard, robust ligand systems that can maintain the molecular integrity of catalysts and reduce the overpotential of WO to levels suitable for industrial application are of great interest. Over the past four decades, catalyst design has evolved significantly, in terms of both efficiency and stability.

**Figure 1:**
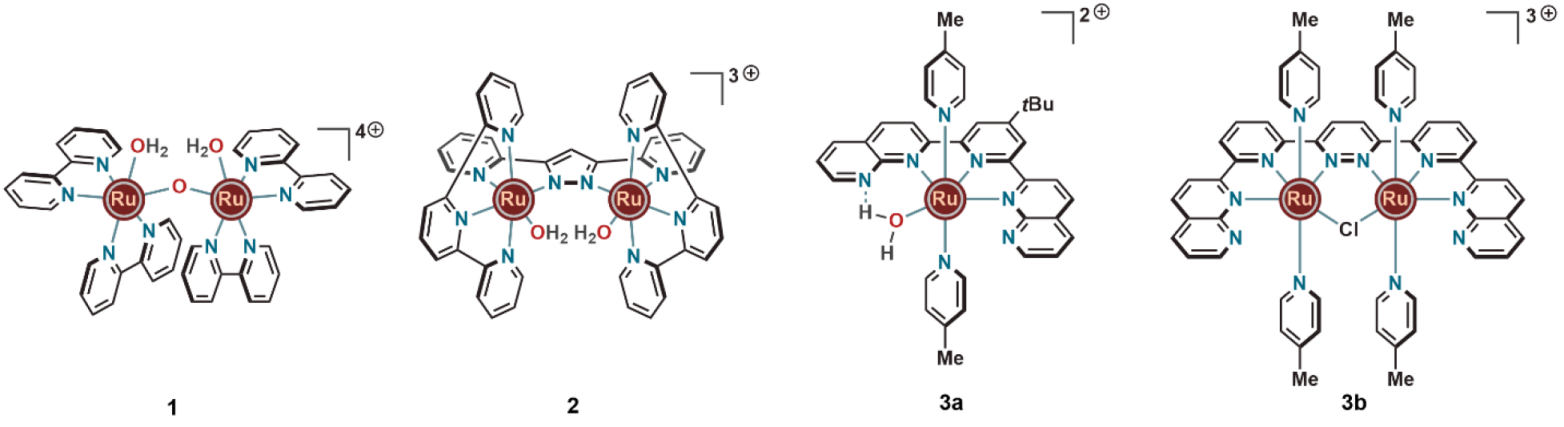
First examples of ruthenium complexes showing catalytic water oxidation activity.

The first study on WO promoted by a molecular transition-metal complex was reported in 1982 by Meyer and co-workers.^8^ The oxo-bridged dinuclear ruthenium(III)-aqua complex (**1**) (Figure 1) equipped with bipyridine ligands was reported to involve a ruthenium(V)-oxo species as the key catalytic intermediate. It was proposed that the O–O bond formation mediated by this catalyst proceeded via either (i) intramolecular coupling between two Ru(IV)-oxyl or Ru(V)-oxo groups (I2M type mechanism) or (ii) nucleophilic attack of water on the electron deficient Ru(V)-oxo unit (WNA type mechanism). However, the stability and efficiency of this catalyst were low, as it exhibited a turnover number (TON) of merely 13.2 and a turnover frequency (TOF) of 4.2 × 10^–3^ s^–1^ for Ce^IV^-driven (from ceric ammonium nitrate) chemical oxidation of water. Due to the catalyst’s decomposition and deactivation during WO, the Faradaic efficiency was only around 19% for a bulk electrolysis at 1.62 V (vs NHE^a^ at pH 1).^9^

Following this pioneering work, Collin and Sauvage highlighted the importance of having a dinuclear framework for achieving water oxidation. Studying Ce^IV^-induced oxidation of a series of mononuclear ruthenium complexes containing 6,6′-dimethyl-2,2′-bipyridine and 2,9-dimethyl-1,10-phenanthrolin ligands, they showed that only **1** with a dinuclear framework was catalytically active in WO.^10^

It took until 2004 before Llobet and co-workers established that the presence of an oxo bridged Ru–O–Ru motif was not really essential for a catalyst to display WO activity.^11^ Instead, a close proximity (for O–O bond formation) of the two ruthenium centers was deemed important, as demonstrated by their catalyst, featuring a 3,5-di(2-pyridyl)pyrazole (DPP) unit as the central ligand backbone and a 2,2′:6′,2″-terpyridine (tpy) unit as the coligand. With this heteroleptic dinuclear ruthenium complex (**2**, Figure 1), a TON of 512 and a TOF of 1.4 × 10^–2^ s^–1^ were reached using Ce^IV^(pH 1) as the chemical oxidant. Without the presence of an oxo bridge, the key O–O bond formation step for catalyst **2** was instead facilitated by a favorable disposition of the two *in situ* generated ruthenium(IV)-oxo units. By having two ruthenium centers rigidly facing each other, the competing anation side reactions that caused catalyst decomposition for complex **1** after merely a few turnovers could be avoided.

In the following year, Thummel and co-workers reported a pyridine–naphthyridine-based mononuclear ruthenium complex **3a** (Figure 1), which demonstrated much lower catalytic activity for WO (TON of 580) than its dinuclear counterpart. With the dinuclear analogue housing 4-picoline as axial ligands (**3b**) (Figure 1), a TON of 3200 was achieved under identical reaction conditions as were used for complex **2**.^12^

Although the demonstration that the complexes **1**, **2**, **3a**, and **3b** catalyzed WO in the presence of Ce^IV^ was an important step forward, the high overpotential required for initiating the catalysis remained as a major obstacle for realizing photocatalytic WO. This reaction represents a promising approach for generating sustainable and economically feasible fuel (hydrogen) from water. To enable photocatalytic WO, photosensitizers such as the ruthenium bipyridine complex **4a** are typically used to regenerate the catalytically active form of the WO catalysts. During the reaction, the photosensitizer is excited by visible light, after which an electron transfer takes place, either to a stoichiometric oxidant or to a photoanode. The oxidized form of the photosensitizer that is formed in this way, in turn, oxidizes the WO catalyst, triggering catalytic turnover.^13^

**Figure 2:**
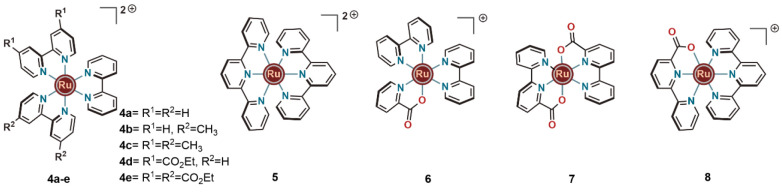
Ruthenium-based photosensitizers and a series of related complexes featuring carboxylate ligands.

When designing efficient photochemical WO schemes, one can aim either (i) to adjust the catalyst design to decrease the potential required for initiating the WO reaction or (ii) to improve the oxidizing ability of the used photosensitizer. In this regard, promoting proton-coupled electron transfer (PCET) with noninnocent ligand systems has turned out to be the most advantageous.^14^ We became interested in this line of research thanks to an earlier study of ours where we found that the introduction of carboxylate groups in the ligand systems of bipyridine ruthenium complexes dramatically decreased the oxidation potentials for the Ru^III^/Ru^II^ couple.^15^ In acetonitrile solutions, both [Ru(bpy)_3_]^2+^ (**4a**) (Figure 2) and [Ru(tpy)_2_]^2+^ (**5**) (Figure 2) have Ru^III^/Ru^II^ redox potentials around 1.54 V. Upon substitution of one of the bipyridines in the former with pyridine-2-carboxylate as in complex **6** (Figure 2), the Ru^III^/Ru^II^ redox potential was decreased to 1.12 V. When two [2,2′-bipyridine]-6-carboxylate units were introduced to form complex **7** (Figure 2), the redox potential was further reduced to 0.76 V. Overall, with this series of ruthenium complexes, a reduction of approximately 0.4 V for the Ru^III^/Ru^II^ couple was observed for each carboxylate group introduced into the ligand system (Table 1). Similarly, replacing one terpyridine unit in complex **5** by bipyridine-2-carboxylic acid resulted in complex **8**, which has a Ru^III^/Ru^II^ redox potential of 1.14 V (Figure 2). This study highlighted the major influence of carboxylate based ligand frameworks in decreasing the oxidation potentials for the Ru^III^/Ru^II^ couple, which constitute the very first step in the catalytic cycle of Ru(II) complexes in electrocatalytic WO.

**Table 1:** Redox Potentials of Ru^III/II^ Couples for **4a**–**e** and **5**–**8**

complex	*E*(Ru^III/II^)_ox_^a^ (V)	solvent	vs	ref
**4a**^b^	1.55/+1.51	MeCN	NHE	(15, 16)
**4b**	1.47	MeCN	NHE	(16)
**4c**	1.43	MeCN	NHE	(16)
**4d**	1.40	H_2_O pH 7.2	NHE	(1)
**4e**	1.54	H_2_O pH 7.2	NHE	(1)
**5**	1.54	MeCN	NHE	(15)
**6**	1.12	MeCN	NHE	(15)
**7**	0.76	MeCN	NHE	(15)
**8**	1.14	MeCN	NHE	(15)

a Converted to NHE (where needed) by adding 0.24 V to potentials vs SCE or by adding 0.63 V for potentials vs Fc/Fc^+^.

b In phosphate buffer (pH 7.2), *E*(Ru^III/II^)_ox_ changes to 1.26 V vs NHE^1^

There are several ways by which the carboxylate group in the ligand framework can influence WO, primarily by (a) stabilizing high-valent oxidation states of the metal center, (b) facilitating PCET processes, (c) maintaining a strong coordination to the metal center that improves the stability of the catalyst, and (d) pH and potential dependent *in situ* decoordination to facilitate incoming water molecules. In this Account, we present the evolution of the carboxylate containing water oxidation catalysts and the conceptual advances that were pivotal to this development. It is worth mentioning that a few complexes can become electrocatalytically inactive due to overstabilization by the carboxylate units. Thus, balancing stability and reactivity in a catalyst is indeed a challenge.

## 2 Evolution of Carboxylate-Containing Ligands in WO Catalysts

**Figure 3:**
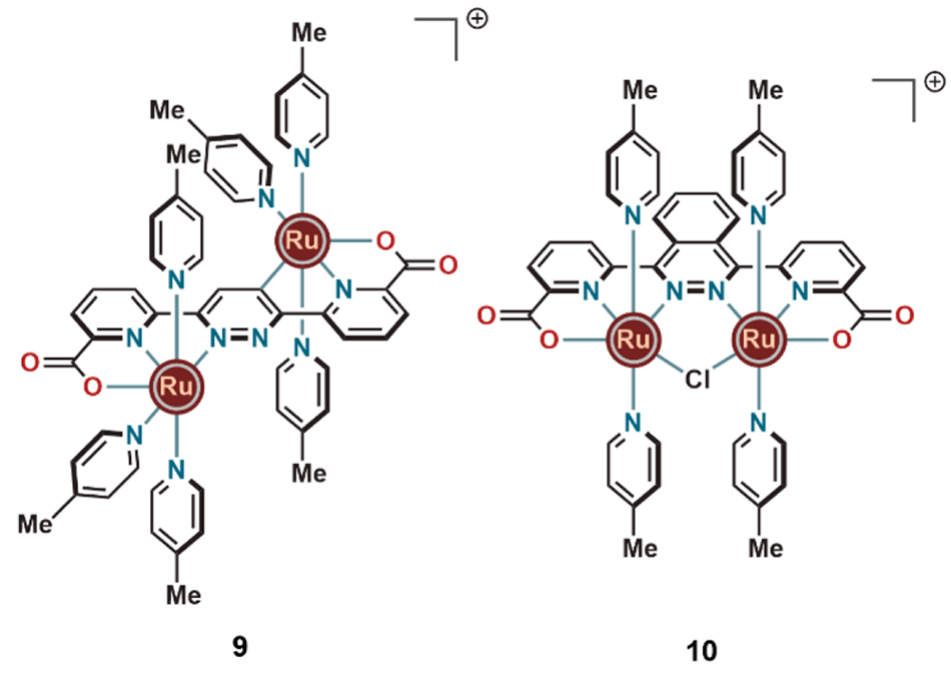
Orientation of the ruthenium centers in two catalytically active ruthenium complexes.

The positive influence of the carboxylate ligand in the first coordination sphere on the Ru^III^/Ru^II^ redox potentials of the above-mentioned ruthenium complexes **6**–**8**, as well as the WO activity of Thummel’s dinuclear catalyst (**3b**), encouraged us to prepare different dinuclear ruthenium-based catalysts featuring carboxylate-based ligand frameworks. The pyridazine–dipyridine carboxylate-based ligand gave rise to complex **9** (Figure 3) with an unexpected *trans*-configured structure of the two Ru centers arising from a C–H activation process at the bridging pyridazine moiety.^17^ When driven by Ce^IV^, complex **9** displayed impressive water oxidation activity, with an observed TON of 4740 and a TOF of 0.28 s^–1^. Although the Ru^III^/Ru^II^ redox potential of complex **9** at pH 7 was found to be considerably lower than that of the previously reported complexes with neutral ligands, the standard photosensitizer **4a** was still not strong enough to induce photochemical WO with **9** as the catalyst. However, by using analogous photosensitizers with slightly better oxidizing properties [e.g., **4d** (E(Ru^III/II^)_ox_= 1.40 V at pH 7.2) and **4e** ( E(Ru^III/II^)_ox_= 1.54 V at pH 7.2)] (Figure 2), photochemically driven water oxidation with TON up to 1300 could be achieved in the presence of persulfate as the sacrificial electron acceptor.^18^ These results indicated ample opportunities for strategical improvements of the ligand framework for both the WO catalysts and the photosensitizers.

Replacing the central pyridazine moiety in **9** with phthalazine made it possible to circumvent the formation of a cyclometalated ruthenium complex via C–H bond activation. Instead, the expected dinuclear Ru complex (**10**) with two ruthenium units in *cis*-configuration bridged by a chloride ligand was obtained.^1^ This new structure was found to significantly improve TON and TOF values for Ce^IV^-induced water oxidation at pH 1. After careful optimization of various reaction conditions, we managed to achieve an impressive TON of 10400 and a TOF of 1.2 s^–1^.

The potential required to initiate the WO by catalyst **10** was suitable to allow photogenerated [Ru(bpy)_3_]^3+^ to drive water oxidation under neutral conditions in the presence of persulfate as the sacrificial electron acceptor, resulting in TON 60 and TOF 0.1 s^–1^. The use of the more oxidizing photosensitizers **4d** and **4e** resulted in improvement of both the TON (420 and 580, respectively) and TOF (0.77 and 0.83 s^–1^) for light-driven water oxidation catalyzed by **10**.^1^

**Figure 4:**
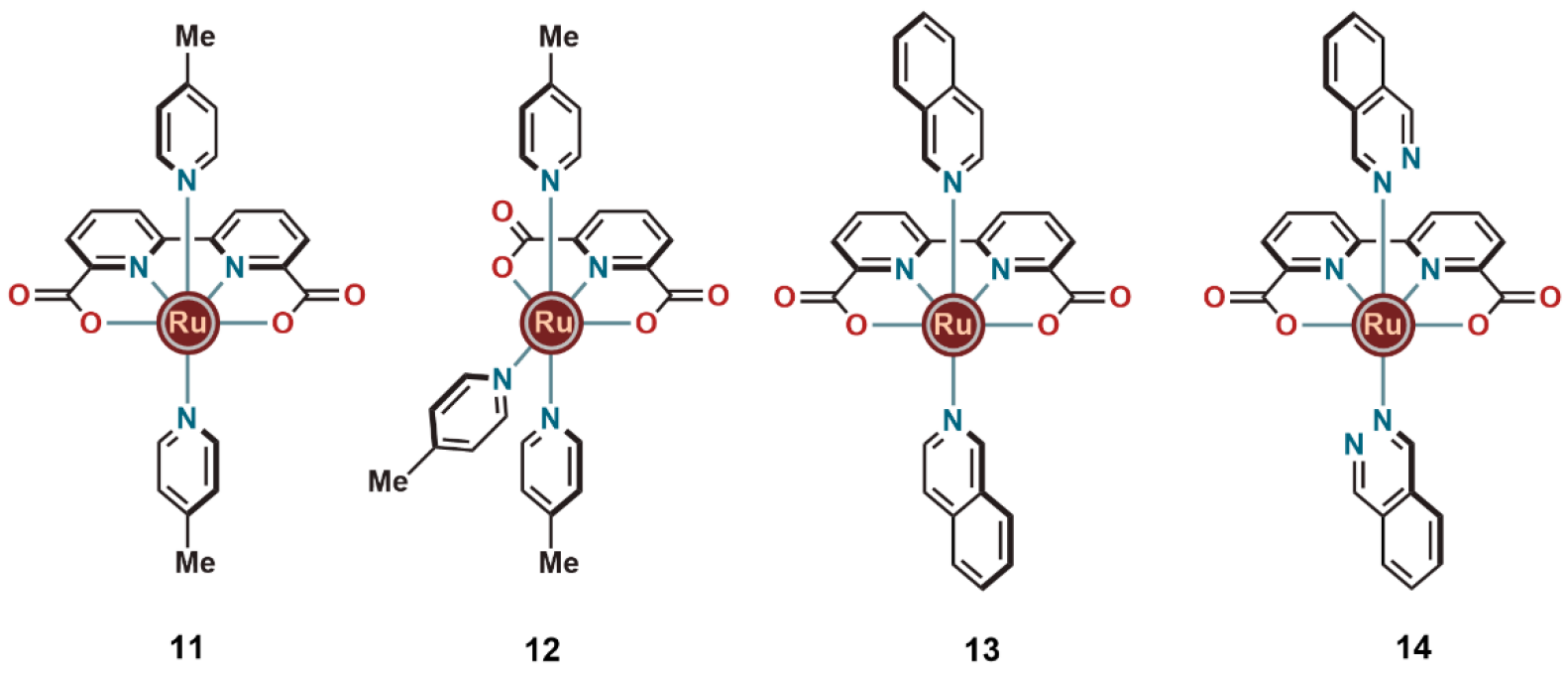
Ruthenium complexes with bda and pda ligands with improved WO activity.

Extending the series of carboxylate-containing ligands, Sun and co-workers reported a mononuclear catalyst **11**, featuring a 2,2′-bipyridine ligand with two carboxylate substituents. Complex **11**, based on the 2,2′-bipyridine-6,6′-dicarboxylate (bda) ligand, having 4-methylpyridines as axial ligands (Figure 4) was reported to mediate water oxidation through a seven-coordinated reactive Ru^IV^ dimer with a bridging [HOHOH]^−^ unit.^19^ Interestingly, it was found to incorporate two hydrogen bonded water molecules near to the active site, and it mediated WO through the I2M type mechanism. This was one of the first reports that included structural characterization of the semistable WO intermediate, highlighting the influence of the carboxylate units in the ligand to stabilize seven-coordinated reaction intermediates.

In 2010, Sun and co-workers reported a related complex (**12**) (Figure 4), in which the bda ligand of complex **11** had been replaced by a pyridine-2,6-dicarboxylate (pda) scaffold.^20^ Due to the different structures of the ligands, the O–Ru–O bite angle in complex **12**, is significantly smaller than that of **11** (157° vs 237°). The smaller bite angle in **12** was proposed to be beneficial for WO, and it was reported to be among the most efficient WO catalysts at that time in terms of TON and TOF (550 and 0.23 s^–1^ with Ce^IV^ as the chemical oxidant). In addition, complex **12** was capable of oxidizing water photochemically using [Ru(bpy)_2_(deeb)]^2+^ [deeb = diethyl (2,2′-bipyridine)-4,4′-dicarboxylate)] as the photosensitizer and persulfate as the sacrificial electron acceptor. Furthermore, the authors found that the activity could be significantly improved by replacing the methyl groups in the axial 4-picolines of complex **12** with stronger electron-donating methoxy functionalities.^21^

In 2012, Sun and co-workers revisited the 2,2′-bipyridine-6,6′-dicarboxylic acid (bda) framework and replaced the axial ligands (4-picoline) of **11** with two isoquinoline units (**13**) (Figure 4).^22^ The intermolecular π-stacking interactions between the isoquinoline units promoted the radical coupling between the Ru^V^=O intermediates, which facilitated O–O bond formation and avoided steric hindrance from the methyl units of the 4-picolines that was present in their previous complex **11**. This resulted in an impressive TON of >8000 and TOF of >300 s^–1^ in Ce^IV^-induced water oxidation. This study was further extended by utilizing a series of other axial ligands (pyridazine, pyrimidine, and phthalazine), keeping the central dicarboxylate Ru-bda unit intact.^23^ Of these, the catalyst with two axial phthalazine ligands (**14**, Figure 4) displayed the highest WO activity (with Ce^IV^ as the stoichiometric oxidant), affording a TON and initial TOF of 55400 and 286 s^–1^, respectively.

**Figure 5:**
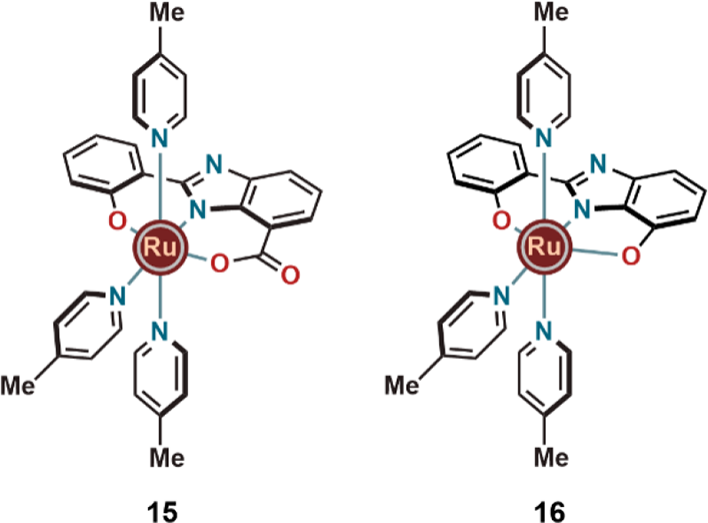
Ruthenium complexes that showed augmented WO activity in the presence of a carboxylate-containing ligand.

In the same year, our group presented two mononuclear ruthenium complexes containing tridentate benzimidazole-phenol based equatorial ligands with either a COOH (**15**) or OH substituent (**16**) on the benzimidazole unit (Figure 5).^2^ Both of the ligands furnished mononuclear ruthenium complexes with 4-picolines as the auxiliary ligands. The clear difference in WO activity with more than 20-fold increase in TON (4000 and 180 for **15** and **16**, respectively) for the carboxylate containing catalyst (**15**) confirmed the positive impact of having a carboxylate group in the first coordination sphere.

**Figure 6:**
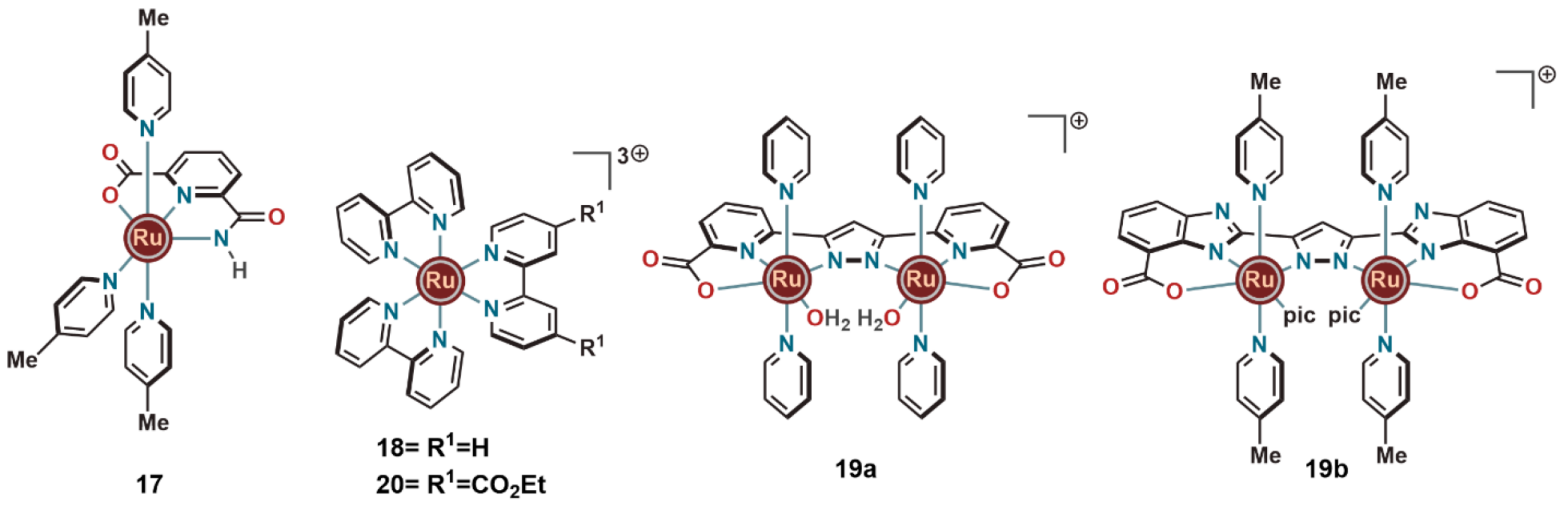
Ruthenium complexes (**17**, **19**) that showed improved electrocatalytic WO activity and could induce WO in the presence of a mild one-electron oxidant (**18** and **20**).

In 2014, our group reported that the strongly electron-donating and chelating properties of 6-carbamoylpicolinate (Figure 6) enabled the formation of the highly a robust Ru^III^-based WO catalyst (**17**) having three 4-picolines as the auxiliary ligands.^24^ The formation of a stable Ru^III^–carboxamide complex confirmed that 6-carbamoylpicolinate is a stronger electron donor than the pyridine-2,6-dicarboxylate ligand.^20^ In a neutral phosphate buffer solution, complex **17** was capable of oxidizing water in the presence of the mild one-electron oxidant [Ru(bpy)_3_]^3+^ (**18**) and achieving a TON and TOF of 280 and 1.6 s^–1^ respectively.^25^ This implied that **17** can be potentially used as catalyst for photocatalytic water oxidation. Under similar electrochemical WO conditions as were used for **11**–**16**, significantly lower overpotential for WO was displayed by **17**.

In 2015, the impact of carboxylate units and noninnocent ligand scaffolds on WO was further investigated by the Meyer and Llobet groups using the dinuclear complex **19a** and by us using complex **19b** (Figure 6). These dinuclear ruthenium complexes were built from pyrazolate–pyridine carboxylate and pyrazolate–benzimidazole carboxylate based ligand frameworks, respectively.^26,27^ Complex **19a** was reported as one of the most active WO catalysts at that time in electrochemically (low WO overpotential) and chemically (Ce^IV^-induced, pH 1, TOF of 8.6 s^–1^) driven water oxidation.

At that time, our group was working on improving the performance of complex **9** by replacing its central pyridazine unit by a pyrazole and the pyridine carboxylic acid core by a benzimidazole carboxylic acid. This afforded the dimeric complex **19b** with its two ruthenium centers arranged in a *cis* orientation.^27^ These changes decreased the overpotential of WO by a considerable extent (onset potential 1.20 V at pH 7.2), demonstrating the positive effect of the two *cis* orientated ruthenium centers. The pyrazole–benzimidazole, being redox noninnocent ligand systems, also helped to stabilize the higher oxidation states of the metal center in the active form of the catalyst, enabling complex **19b** to oxidize water both under chemical (using the mild oxidant **18** with a TON of 800 at pH 6.2) and photochemical conditions. Furthermore, when [Ru(bpy)_3_]^2+^ (**4a**) was replaced with [Ru(bpy)_2_(deeb)]^2+^ (E(Ru^III^/Ru^II^)ox = 1.40 V vs NHE; deeb = diethyl (2,2′-bipyridine)-4,4′-dicarboxylate) (**4d**) (Figure 2), the photocatalytic activity could be further improved, resulting in a TON of 890 at pH 6.2.

**Figure 7:**
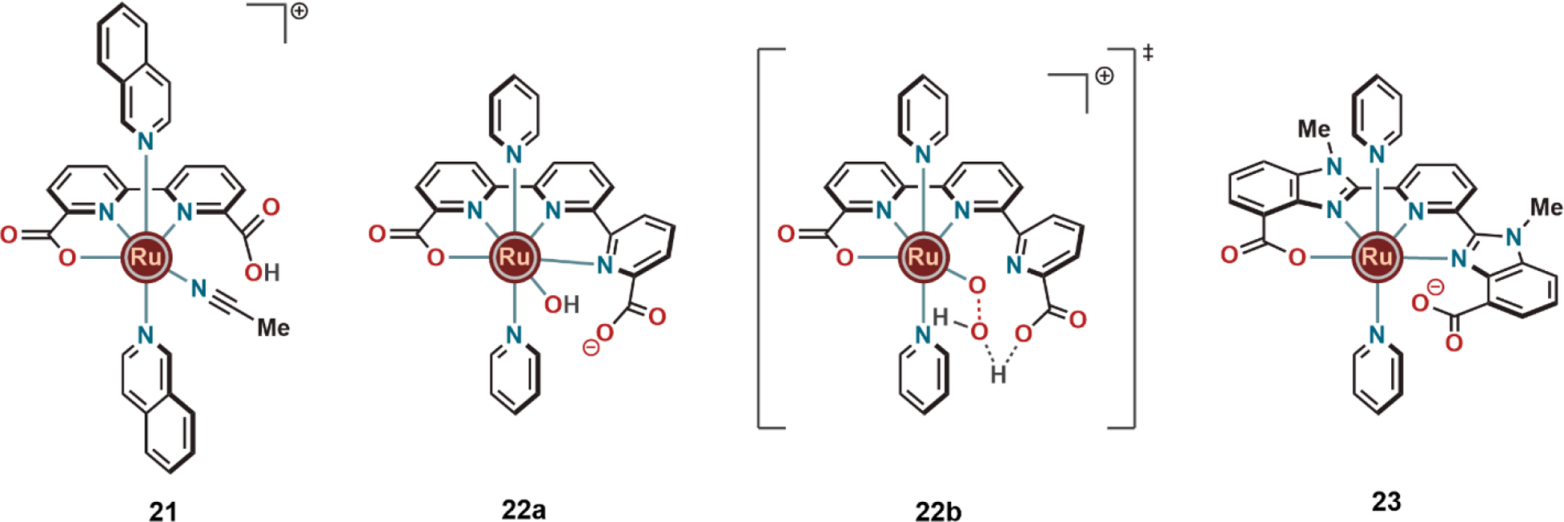
Hemilabile and labile carboxylate groups in the ruthenium complexes.

The hemilability of the ligand carboxylates is an interesting feature that has been reported to have a strong positive influence on the WO activity of several Ru complexes. In 2015, Meyer and co-workers studied the Ru-bda complex **13** at pH ≥ 2.4 with 4% (v/v) MeCN and confirmed the hemilability of one of the carboxylate units by the crystal structure (**21**) as well as by ^1^H and ^13^C NMR spectroscopy (Figure 7).^28^ The rate of WO using this open arm chelate (**21**) could be significantly improved by the addition of proton acceptors, such as AcO^–^, HPO_4_^2–^, PO_4_^3–^, and OH^–^. At pH 7, it was found that the higher concentration of H_2_PO_4_^–^/HPO_4_^2–^ also had a positive impact on the WO rate. The reaction was proposed to go through either an atom–proton transfer or a concerted electron–proton transfer pathway. Similar displacement of the carboxylate group by MeCN cosolvent was also observed for other WO catalysts, including the Ru(pda)-derived catalysts described by Sun and our group.^20,29^

The positive influence of the dangling carboxylate group on catalytic WO was also recognized by Llobet and co-workers, who studied the ruthenium complex **22** housing [2,2′:6′,2″-terpyridine]-6,6″-dicarboxylate (tda) as the equatorial ligand and pyridines as the axial ligands (Figure 7).^30^ The O(carboxylate)–Ru–O(carboxylate) bite angle toward the central terpyridine unit was 291°, which was much higher than that of complexes **11** and **12**. The hydroxy species [Ru^IV^(OH)(tda)(py)_2_]^+^ (**22a**) (Figure 7) displayed an impressive TOF_max_ of 8000 s^–1^ (as measured by the foot of the wave analysis of the electrochemical WO) in phosphate buffer at pH 7.0. This was about 3–4 orders of magnitude higher than that of [Ru(bda)(pic)_2_] measured at pH 1 with Ce^IV^ as oxidant. The improvement of the WO activity was explained by a PCET mechanism involving the dangling carboxylate ligand that acts as a proton acceptor for the incoming water molecule and facilitates O–O bond formation with the Ru(IV)-oxyl radical intermediate (Figure 7).

**Scheme 2:**
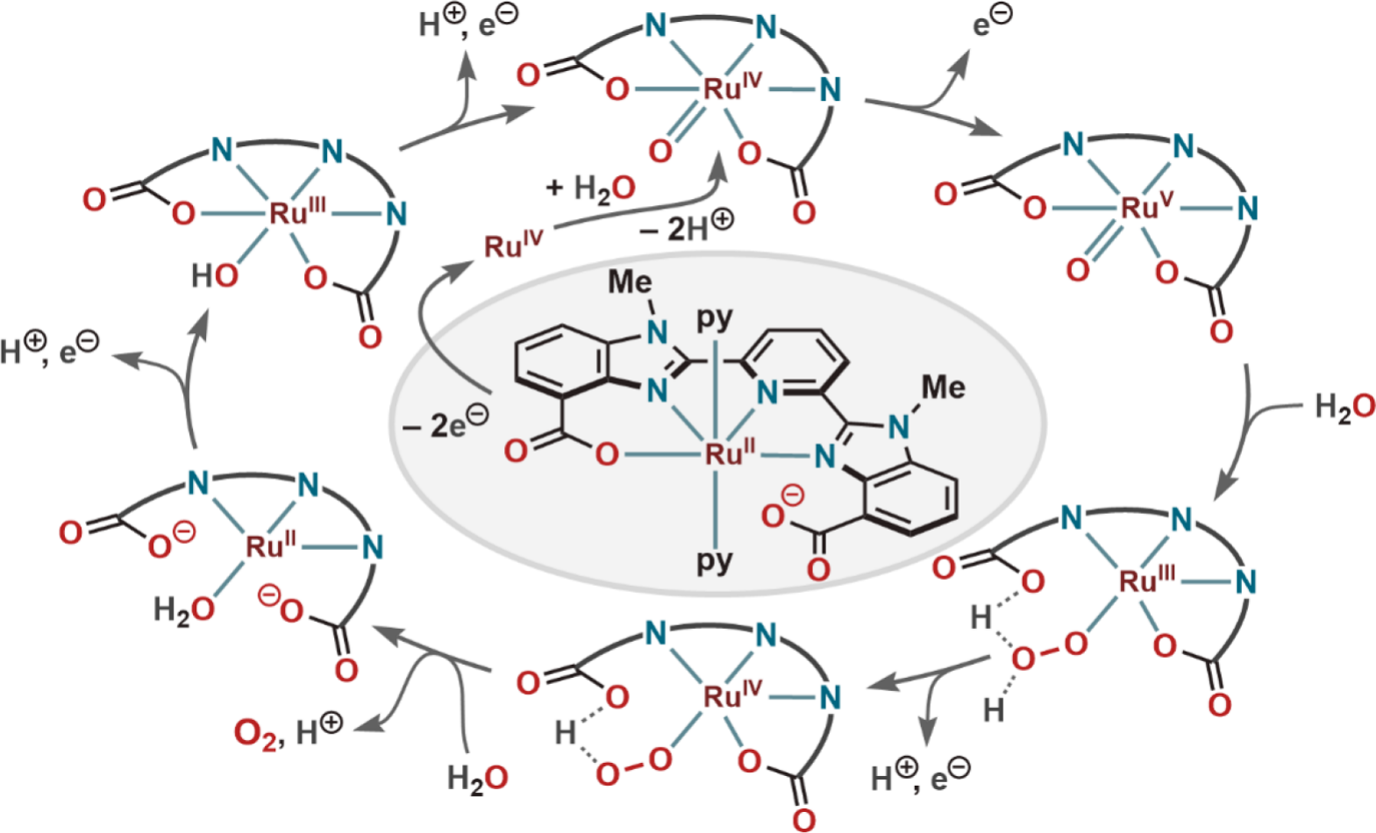
Proposed Catalytic Cycle for WO by Ru-Complex (**23**) Having a Noncoordinated Ligand Carboxylate Unit in the Ground State

The reactivity of complex **22** was further improved by our group by exchange of the central pyrazole of **19b** by a pyridine (mcbp^2–^) unit and monomethylating the benzimidazole units to form **23**. This mononuclear Ru(II) complex (**23**) (Figure 7) having one dangling carboxylate unit from the ligand and two pyridines as axial ligands showed a TOF_max_ of 40000 s^–1^ in the foot of the wave analysis of the electrochemical WO process at pH 9.^4^ The carboxylate group in the close vicinity of the metal center was proposed to be involved in PCET (Scheme 2). Moreover, an intramolecular atom proton transfer (*i*-APT) from the incoming water molecule to the carboxylate oxygen (as for **22**) lowered the activation barrier for the rate limiting O–O bond formation step. The WO process was proposed to proceed through a Ru^III^(Hmcbp)(O–OH) transition state that was formed by water nucleophilic attack on the [Ru^V^(mcbp)(O)] intermediate.

**Figure 8:**
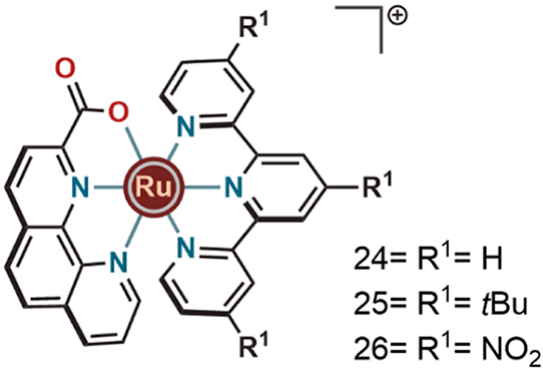
Phenanthroline carboxylate-based Ru complexes for WO.

Continuing along this line of research, Thummel’s group and our group reported three ruthenium complexes (**24**, **25**, and **26** in Figure 8) with very similar ligand frameworks that were active in Ce^IV^ and electrochemically induced WO.^3,31^ These heteroleptic complexes had a phenanthroline carboxylate (phc) unit and terpyridine as ligands. These studies showed that improving the catalytic WO efficiency through the incorporation of hemilabile or dangling carboxylate units was not only limited to the pyridine and benzimidazole based ligand frameworks, but instead that it is a much more general trend that is applicable to many other kinds of robust ligand frameworks as well. Electron donating groups on the terpyridine ligand help to achieve the Ru^III^ state and hence decrease the redox potential of the Ru^III/II^ (**26** > **24** > **25**) couple (Table 2). In the case of complex **25**, it was reported that the phc ligand not only helps to maintain the molecular integrity of the catalyst, but also assists by participating as a redox-active and pH dependent hemilabile site. It also provided the required space for the incoming water molecule and helped to stabilize the higher oxidation states.

**Table 2:** Redox Potentials of Ru^III/II^ Couples vs NHE for **24**–**26**

complex	*E*(Ru^III/II^)_ox_^a^ (V)	solvent	vs	ref
**24**	+1.06	MeCN	NHE	(30)
**25**	+0.99	MeCN	NHE	(3)
**26**	+1.17	MeCN	NHE	(3)

a Converted to NHE by adding 0.24 V to potentials vs SCE or by adding 0.2 V for potentials vs Ag/AgCl.

Here, electron donating (-*t*Bu) substituents on the terpyridine unit in **25** were found to promote the lability of the carboxylate unit, thereby improving the electrocatalytic WO activity further.^3^

## 3 Theoretical Understanding of the Carboxylate Influence on WO

DFT studies on molecular WO catalysts can provide valuable insight into the reaction mechanism and potential catalytic intermediates. A clear understanding of the electronic and structural features will facilitate the design of the next generation catalysts and help to reduce the amount of experimental work. In 2010, our group together with Privalov and co-workers investigated the seven-coordinated Ru-bda containing WO intermediates and corresponding binuclear reaction (I2M) pathway for O_2_ production.^19,32^ We were able to show that WO proceeded through a radical coupling between two Ru^IV^(bda)–O^•^ species as the rate-limiting step followed by a less energy demanding detachment of O_2_ from the peroxo intermediate. Furthermore, with the help of DFT calculations the involvement of coordinatively unsaturated Ru^II^ or Ru^III^ intermediates in the catalytic pathway could be ruled out.

**Scheme 3:**
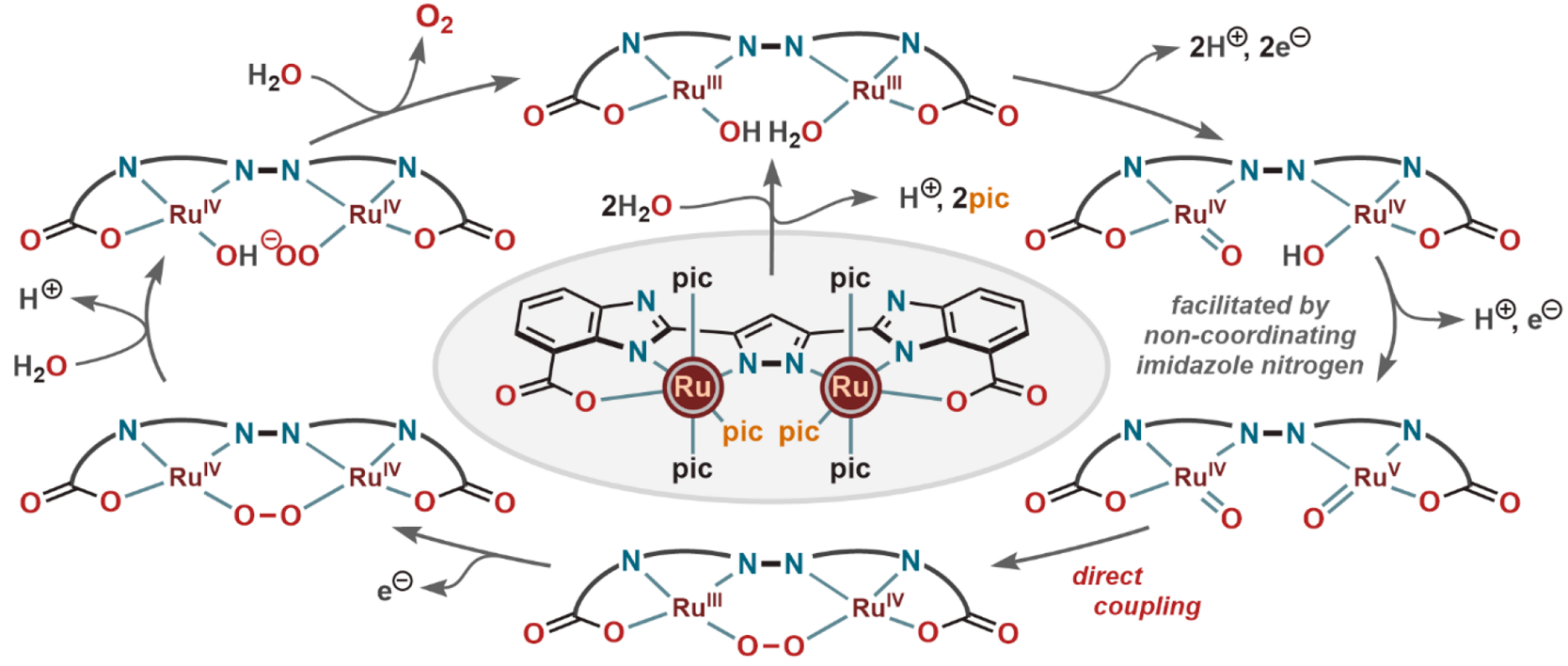
DFT Optimized Proposed Catalytic Cycle for WO by Dinuclear Ru Complex (**19b**)

In 2016, our group together with Siegbahn and co-workers investigated the reaction intermediates and plausible WO mechanisms involving **19b** as the electrocatalyst.^33^ Both the water nucleophilic attack and direct coupling mechanisms were considered at the time. Here, O–O bond formation was energetically favored (by ∼12 kcal mol^–1^) for intramolecular coupling of two *cis*-oriented Ru_2_^IV,V^-oxo units (Scheme 3). Although in most of the reported electrocatalysts, the O–O bond formation is shown to be the rate-limiting step, in the case of **19b**, it is the oxygen release from the Ru^IV^(OH)Ru^IV^(O–O) intermediate that was proposed to be rate-limiting. Throughout this mechanism, carboxylate groups in the ligand framework help to maintain the molecular integrity of the catalyst and reduce the oxidation potential of the redox couples.

**Figure 9:**
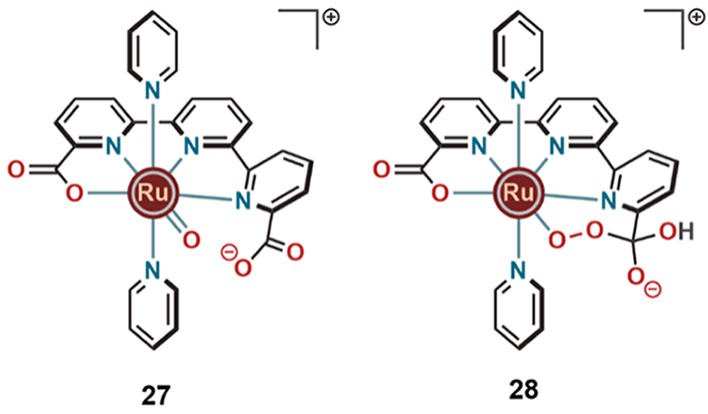
Catalytic intermediates for WO with **22**, showing carboxylate involvement.

To understand the details of WO using **22**, a theoretical investigation of the possible reaction mechanisms was performed by Ahlquist and co-workers in 2019.^34^ Both the low and high pH reaction scenarios were analyzed in corroboration with the experimental results. According to this study, the participation of a noncoordinated ligand (tda) carboxylate in the oxide relay was an essential factor as proposed earlier.^30^ This dangling carboxylate unit can provide an intramolecular nucleophilic oxygen close to the Ru^V^(O) center to facilitate O–O bond formation (**27**) (Figure 9) and can also serve as a remote electrophilic center for the nucleophilic attack from the incoming OH^–^ units (**28**) (Figure 9). The WO reaction does not proceed through a typical WNA to Ru^V^(O) to form the O–O bond. Instead, the percarboxylate intermediate proceeds through C–O bond cleavage resulting in formation of triplet O_2_. At low pH (<7) the nucleophilic attack of OH^–^ constituted the rate limiting step, whereas at high pH (>8), it changed to the O–O bond formation step.

**Scheme 4:**
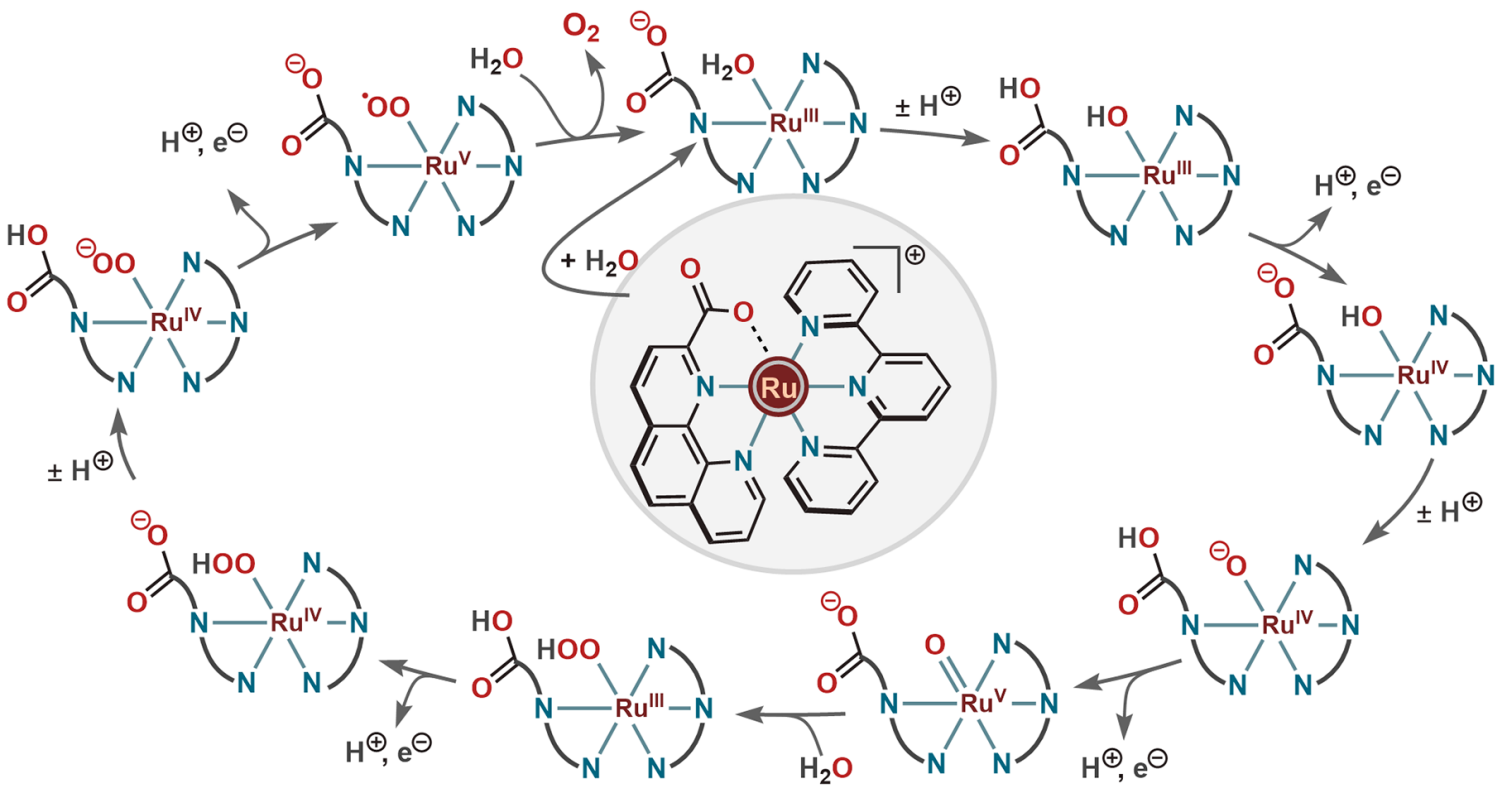
DFT Optimized Proposed Catalytic Cycle for WO by Mononuclear Ru Complex (**24**)

Another DFT investigation of the WO mechanism using **24** as the electrocatalyst was performed by Yan and co-workers in 2020.^35^ Here as well, the authors could find support for the active participation of the carboxylate unit in both O–O bond formation and oxygen release (Scheme 4). In both cases, the carboxylate group acts as a proton acceptor in which it facilitates WNA by decreasing the activation barrier of the nucleophilic attack of the incoming water molecule to the Ru^V^=O species.

The kinetic studies indicated impressive rate enhancements due to the presence of the carboxylate unit, in analogy to the external base that helps most of the WO catalysis. The O_2_ release, which involves loss of two protons and two electrons, also gets enhanced by the proton accepting capability of the carboxylate unit. It does so by effective shuffling of the proton from the Ru^IV^–OOH intermediate, thereby facilitating the release of O_2_.

## 4 Current Trends

In order to improve the reactivity and stability of molecular systems for WO to enable industrial use, an effective and recent trend is to immobilize or anchor these carboxylate containing electrocatalysts onto conductive materials, such as carbon nanotubes, reduced graphene oxide, or TiO_2_, to furnish robust anodes.^36^ Depending on the optimal operating potential of the anodes, they can also be coupled with photosensitizers to develop photoanodes that make use of energy directly from sunlight to initiate WO.

**Figure 10:**
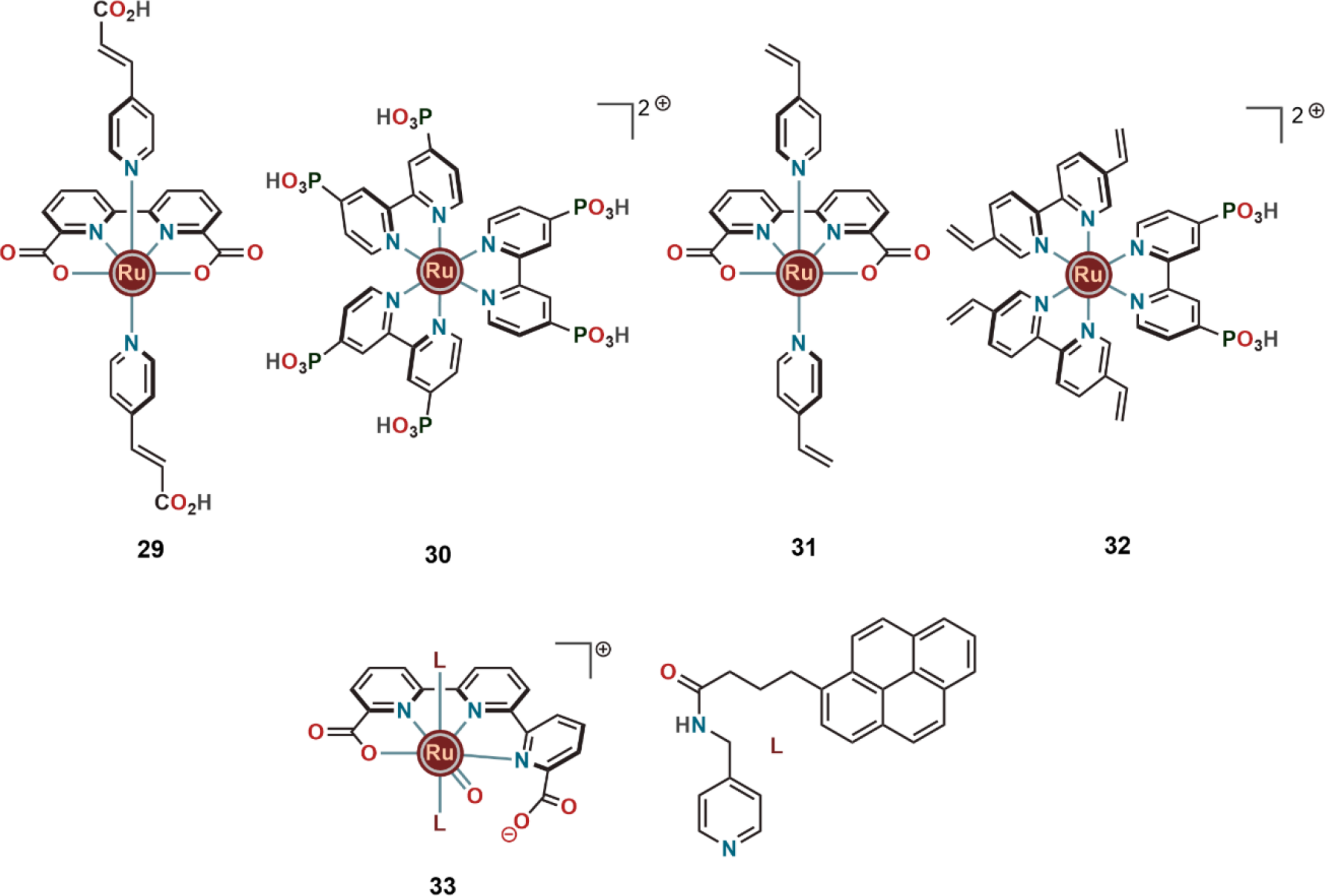
Selection of Ru complexes that were tested on conductive surfaces for improved WO efficiency.

As an example, Sun and co-workers prepared a photoanode using **29** as catalyst by replacing the 4-picoline groups with the 3-(pyridin-4-yl)acrylic acid of **11** and using phosphorylated **4a** [Ru(4,4′-(PO_3_H_2_)_2_bpy)_3_]Cl_2_ (**30**) as photosensitizer (Figure 10). The photosensitizer stayed directly attached to the nanostructured TiO_2_ by the phosphoric acid group that interacted with the electrocatalyst in a supramolecular fashion through Zr^4+^ units.^37^ Subjecting this photoanode to 450 nm light, a current conversion efficiency of 4.1% and a photocurrent density of 0.48 mA cm^–2^ could be achieved in a three-electrode set up. Although the overall efficiency was insufficient for industrial use, this report indicates the possibility of utilizing supramolecular networks for preparing anodes.

Electro-polymerization is another promising technique that was utilized by Meyer and co-workers to link carboxylate containing molecular catalysts to photosensitizers. Vinyl functional groups that undergo electro-polymerization easily were used in both the electrocatalyst (**31**) and the photosensitizer (**32**) (Figure 10).^38^ In this photoanode, the electrocatalyst (**31**) was covalently linked to the photosensitizer (**32**), which was in turn anchored to the mesoporous TiO_2_ through phosphoric acid linkers. From the kinetic studies of the photo-electrocatalytic WO, it was proposed that the coordination chemistry and PCET properties involving the carboxylate group in **11** stayed valid for **31** as well. Even after the electropolymerization, O–O bond formation remained the rate-limiting step.

That the interesting features of the carboxylic acid groups in homogeneous WO catalysts also stay effective upon immobilization or anchoring onto a conductive surface was further supported by Ahlquist and co-workers. Recently, they studied a hybrid system of [Ru(tda)(L)_2_] (L = 4-(pyren-1-yl)-N-(pyridin-4-ylmethyl)-butanamide) (**33**) immobilized onto carbon nanotubes for applications in WO (Figure 10). Their results on electrocatalytic WO suggest that the oxide relay involving the carboxylate group in the molecular electrocatalyst also applies in the hybrid system.^39^ The dangling carboxylate group in the tda ligand could efficiently induce oxide relay in a similar fashion on the carbon nanotubes as discussed above using complex **22** under homogeneous conditions. It was also proposed that strong π-stacking interactions between the pyrene units of the catalyst molecules and the carbon nanotubes helped to reduce the mobility of the catalyst during electrolysis, which led to improvement of the stability and in turn the overall reactivity.

## 5 Conclusions and Outlook

With the aim to generate renewable and environmentally benign fuel (H_2_) efficiently, water oxidation (WO) electrocatalysts have evolved significantly over the past two decades. To attain TON and TOF required for large scale applications, long-term stability and augmented reactivity of the catalysts are of fundamental importance. Here, strategic ligand design plays a crucial role. As discussed in this Account, the carboxylate/carboxylic acid groups in the ligand framework have shown very positive effects in this regard. Incorporating these groups into the ligand scaffold can enable PCET, *i*-APT, redox, and pH-dependent lability. All these phenomena are vital for lowering the overpotential of WO. In addition, being negatively charged, carboxylate groups in the first coordination sphere can directly assist in maintaining the integrity of the catalyst under strongly oxidative conditions, providing a firm grip to the metal center by chelation. The negative charge can also help to lower the activation barrier for generation of high-valent intermediates and positively charged transition states.

**Figure 11:**
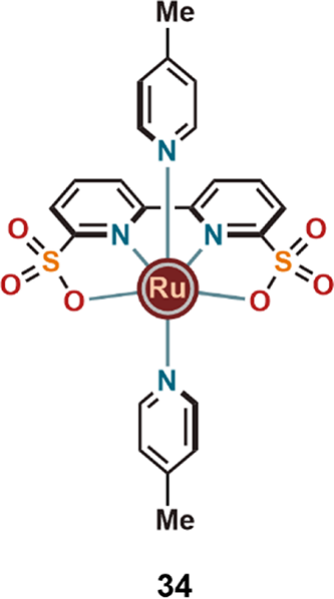
Recently published, closely related Ru complex (**34**) with improved WO activity

We expect to see further structural improvements of WO catalysts through the use of negatively charged and noninnocent ligands and axial coligands. For example, Sun and co-workers have recently demonstrated that the special features of the carboxylic acid/carboxylate groups can also be achieved by ligands carrying sulfonate group donors. Replacement of the two carboxylate groups of the Ru-bda complex (**11**) by sulfonate groups provided complex **34** (Figure 11) with improved TOF values for electrocatalytic WO in both acidic and neutral (160 and 12900 s^–1^, respectively, at pH 1 and pH 7) conditions.^40^ The introduction of halogen substituents and electron-donating groups into the axial coligands of carboxylate containing Ru complexes might also provide catalysts with superior activities.^41^

Utilization of strategically designed carboxylic acid/carboxylate-based ligands for preparation of multinuclear metal complexes in order to exploit the cooperative influence can be regarded as one of the other promising approaches. Our group together with our collaborators are currently working on this.

The impressive ability of these carboxylate/carboxylic acid group containing electrocatalysts to retain their molecular properties even upon anchoring onto conductive surfaces will open up the prospect of grafting these electrocatalysts onto materials, such as carbon cloth, carbon nanotubes, and reduced graphene oxides. This approach has potential to improve current densities and long-term stabilities to meet the standards for industrial use.^42^

## Footnotes

a All potentials reported in this Account are converted to NHE.
